# Editorial: The Global Impacts of COVID-19 on Maternity Care Practices and Childbearing Experiences

**DOI:** 10.3389/fsoc.2021.721782

**Published:** 2021-07-05

**Authors:** Robbie Davis-Floyd, Kim Gutschow

**Affiliations:** ^1^ Department of Anthropology, Rice University, Houston, TX, United States; ^2^ Department of Anthropology, Williams, College, Williamstown, MA, United States

**Keywords:** COVID-19, maternity care, pregnancy, birth, obstetricians, midwives, doulas, childbearing women

## Introduction: Presenting Our Collection and Identifying Salient Themes

This special issue on *The Global Impact of COVID-19 on Maternity Care Practices and Childbearing Experiences* includes articles that describe the experiences of providers and childbearers in relation to pregnancy, childbirth, and the postpartum period during the COVID-19 pandemic across a range of countries, including the United States, Canada, Mexico, Chile, Italy, Russia, India, Pakistan, Kenya, and New Zealand, as well as an article on pandemic doula care across 23 high- and middle-income countries. Most of the articles in this collection primarily examine the COVID-19 pandemic either from the perspective of providers—including midwives, doulas, obstetricians, nurses, social workers, and other birthworkers—or from the perspective of childbearers. We begin this Editorial by focusing mostly on providers, then turn to childbearers’ experiences. All references without dates refer to articles in this Special Issue.

These articles cumulatively emphasize that the coronavirus pandemic has revealed and highlighted deep fragmentations, inequalities, and dysfunctions within maternity care that existed before the pandemic began. Indeed, this pandemic offers both a disruptive moment and a long-overdue opportunity to fix systemic problems within maternity care in ways that can benefit providers, mothers, newborns, and families (Gutschow et al., 2021). In short, the pandemic offers an opportunity to shift maternity care toward justice, equality, and human rights for all, as we will further address in our Conclusion to this Editorial.

## Providers’ Adaptive Responses to Shifting Evidence: COVID-19 and SARS-CoV-2

Risk and fear were major themes for providers working within the rapidly evolving situation of COVID-19, in which basic knowledge about the virus, SARS-CoV-2, and the disease it causes, COVID-19, were rapidly evolving during much of 2020 and 2021. As we illustrate (Gutschow and Davis-Floyd), providers were responding to very limited or unproven “evidence” about routes and risks of transmission, including understanding viral loads; how to estimate and mitigate widespread asymptomatic community transmission; and estimating case fatality rates and the progress of the disease—especially for pregnant people. In the early weeks and months of the pandemic, providers were overwhelmed, and given no or limited evidence about how SARS-CoV-2 or COVID-19 would affect pregnant women, fetuses, and newborns, while misinformation and rapidly shifting protocols—some of which were later withdrawn for lack of evidence—increased the confusion (Gutschow and Davis-Floyd).

Many of our articles indicate what we term *information overwhelm* as a primary stressor for maternity care providers, due to the rapid and unpredictable shifts in protocols, evidence, and guidance. As access to testing, PPE, and evidence about routes of viral transmission and treatment for COVID-19 improved during 2020, providers were able to overcome some early fears and misinformation. In United States hospitals with access to adequate PPE and testing, providers gained better estimates about the risk of contracting COVID-19 at work and the health risks of asymptomatic infections for mothers and newborns with SARS-CoV-2 (Gutschow and Davis-Floyd). In Puerto Rico and Mexico, community-based midwives were held under suspicion of spreading contagion and denied the ability to accompany their transferred clients in hospitals as well as PPE early on, exacerbating existing policies that already denied them official recognition and government support (Reyes; Alonso et al.).

Throughout the pandemic, providers committed to women’s agency and humanistic birth have needed to be nimble in absorbing new information about SARS-CoV-2 and COVID-19, while adapting their protocols and practices in ways that protect their fundamental approach to birth. Midwives and birthworkers in a Chilean hospital (Leiva et al.), New Zealand (Crowther et al.), Canada (Rudrum; Daviss et al.), in the Luna Maya birth centers of Mexico (Alonso et al.), and in some places in the United States (Gutschow and Davis-Floyd; Oparah et al.; Rivera) were able to provide respectful and humanized maternity care. Many providers struggled to push for holistic and humanistic models of care while limiting unnecessary interventions and cesareans (Gutschow and Davis-Floyd; Daviss et al.).

In March of 2020, the International Confederation of Midwives (ICM) stressed the need for midwives to be recognized as essential workers, yet countries like Mexico and the United States have failed to integrate community-based midwifery care into their respective maternity care systems (Alonso et al., Reyes; Gutschow and Davis-Floyd). While freestanding birth centers in Mexico, Puerto Rico, and New Zealand continue to provide humanized care that respects women’s autonomy and decisions around birth plans and partners, they still face increased scrutiny and suspicion from the medical establishment according to their degree of integration into the formal healthcare system (Alonso et al.; Reyes; Crowther et al.).

Many of our articles describe the ways in which fear, bureaucratic or institutional control, absence of oversight, and the absence of labor support people have led to an increase in obstetric violence and/or interventions during the pandemic. While Reyes notes that for Puerto Rico, “some women are coming out of their pandemic hospital births more traumatized than ever…there are many stories of violent deliveries,” the rise in pandemic-related obstetric violence (see Sadler et al., 2016; Liese et al., 2021) is not yet quantified. Doulas in many countries reported a cascade of interventions and mistreatment for clients who were denied a labor support person (see Searcy and Castañeda; Reyes; Rivera; Oparah et al.). As Rivera notes, awareness of cases of preventable maternal deaths for women of color in the United States during the COVID-19 pandemic prompted birthworkers of color to push harder to advocate for their clients’ rights to have support people during labor.

## The Principles of Separation and Prohibition

The articles in our collection show wide variation across nations and regions in the rules specifying how long or under what conditions the labor support person could or had to stay in the hospital. In Canada, one labor support person was allowed, yet restrictions on support people in the neighboring United States made Canadian childbearers nervous (Rudrum). In New Zealand and the United States, restrictions on labor support people were eventually lifted (Crowther et al.; Gutschow and Davis-Floyd; Oparah et al.). In Russia, formal restrictions against labor support persons led people to seek paid contracts that allowed such partners (Ozhiganova). In some Italian hospitals, according to Benaglia and Canzini, labor support persons were initially only allowed during the pushing phase—a restriction that was lifted by the end of April 2020. In some United States hospitals, labor support persons were permitted from the time labor began, as long as they did not leave the hospital, while in other hospitals and countries, they were ordered to leave immediately after the birth (Searcy and Castañeda; Gutschow and Davis-Floyd). Several articles describe childbearers feeling isolated, alone, and traumatized by these injunctions and restrictions against labor support people (Gildner and Thayer; Reyes; Ozhiganova; Oparah et al.; Gutschow and Davis-Floyd; Crowther et al.)

As Benaglia and Canzini describe, the fight for humanized maternity care in Italy runs counter to the tendency for the COVID-19 pandemic to reinforce two technocratic principles. These authors describe both the *principle of separation* and the *principle of prohibition* brought to light by the pandemic:

Hospital spaces, protocols, and hierarchies do rest on the principle of separation, which is complementary to what we are calling the *principle of prohibition*. The biomedical choice to remove the birth partner from the birth scene shows that both principles were amplified in practice during the peak of the crisis.

Drawing on Davis-Floyd (2001), Davis-Floyd (2018a) argument that the technocratic model of birth is based on the fundamental principle of separation, Benaglia and Canzini demonstrate that COVID-19 reinforces the principle of prohibition in medicine, whereby the power of hospitals and providers is structurally related to their power to prohibit. They argue that the principle of separation during childbirth, newborn care, and breastfeeding represents “conceptual and biological nonsense,” given the obvious difficulty of separating newborns from mothers during these vulnerable moments. Benaglia’s and Canzini’s illustration of how Italian hospitals were quick to ignore women’s agency and rights to labor support companions echoes a homebirth obstetrician in the United States, who noted how quickly hospital-based providers abandoned humanized birth models at the start of the COVID-19 pandemic (Gutschow and Davis-Floyd). Benaglia and Canzini close by describing a pervasive fear among midwives that the culture of uncertainty during the pandemic will further normalize the medicalization of birth in Italy. This same fear in other countries is described in Gutschow and Davis-Floyd; Reyes; Alonso et al.

In Chile, as in Italy, bans on labor support people were quickly established and then undone after considerable pushback by childbearers and providers, who argued that such bans were not based on evidence. In one Chilean hospital, La Florida, dedicated to humanized maternity care, labor support companions and skin-to-skin contact between mothers and newborns were banned but then reinstated after only 20 days, because they went so against the grain of that hospital’s highly humanistic model (Leiva et al.).

The injunction against labor support persons leaving the labor room was difficult for those with small children at home or jobs without flexibility, especially given the hardships of finding and/or being able to afford childcare during the pandemic (Gutschow and Davis-Floyd). Many of the articles in our collection note that the hospital policies of separation seem both irrational and arbitrary, as the doula and partner are with the laboring woman right up until she enters the hospital and will accompany the childbearer and newborn as soon as they leave the hospital (Searcy and Castañeda).

## Separation and Prohibitions on Doulas Attending Hospital Births

Across the globe, medical bureaucracies rushed to exclude and erase doulas from labor rooms, to which they had only recently gained access (Searcy and Castañeda). The speed and ease with which medical institutions and providers appeared to neglect the considerable evidence proving the benefit of doulas in providing continuous labor support was shocking (Gutschow and Davis-Floyd). In many countries, doulas struggled for access to labor rooms, for recognition as “essential” or frontline workers, for access to testing and PPE, and for ways to support their clients virtually during labor and delivery if they were denied physical access (Searcy and Castañeda; Reyes; Rivera; Oparah et al.; Gutschow and Davis-Floyd).

In the United States, doulas fought to regain access to hospitals after being banned outright in the early months of the pandemic, while struggling to adapt to constantly changing rules about who was allowed in the labor room or during the postpartum period (Oparah et al.; Rivera; Gutschow and Davis-Floyd). Some United States-based doulas ended up teaching their clients’ partners critical doula skills when it became clear that hospitals would not accept both partner and doula in the labor room but were forcing women to choose between them. In South Africa, restrictions that banned travel for all people except “essential” workers led some doulas to find creative ways to hastily produce doula certificates or special permissions for attending clients (Searcy and Castañeda). In many countries, virtual doula support *via* phone or video chat for antenatal and intrapartum care has become the norm, even as both doulas and clients feel that this is unsatisfactory and detrimental to the labor and birth experience (Searcy and Castañeda; Oparah et al.; Rudrum; Rivera).

## Separation of Mother and Baby

The principles of separation and prohibition were also evident in the forced separation of mother and newborn. After an early recommendation (Favre et al., 2020) that newborns be separated from mothers testing positive, by the summer of 2020, WHO, the CDC, and the AAP (American Association of Pediatrics) all recommended that mothers and newborns be kept together, even if the mother tested positive for SARS-CoV-2, as long as she was not critically ill (see Gutschow and Davis-Floyd). While later studies confirmed a very low risk of transmission from mothers to newborns and evidence that most newborns testing positive for SARS-CoV-2 recovered quickly or were asymptomatic, the damage has been done in many countries where immediate skin-to-skin contact between newborns and mothers has been interrupted or banned.

In the United States, skin-to-skin contact was discouraged or prohibited in one out of five hospitals by the summer of 2020 (Gutschow and Davis-Floyd), and in Russia, all mothers were denied contact with their newborns for at least two weeks (Ozhiganova). While the Russian obstetricians Ozhiganova interviewed thought it was a “terrible measure,” they were “soldiers in a system” that had reverted to an earlier Soviet style, which had emphasized prohibition, separation, bureaucratic paternalism, and neglect of patient rights. A Russian joke captured the fear of overly restrictive measures in maternity wards: “In Russia, the coronavirus is not as terrible as the fight against it!” (Ozhiganova).

New Zealand took a more enlightened approach by ensuring that skin-to-skin contact and breastfeeding have always been supported for all mothers, even those who test positive for the virus (Crowther et al.). In Canada, midwives in First Nations communities worked with local leaders to maintain prenatal visits even during lockdowns, and most especially in Ontario, according to Daviss et al., midwives worked to protect vital skin-to-skin contact between mother and newborn. In Totonicapán, Guatemala, traditional midwives/*comadronas*, who had formerly been welcome to accompany their clients during hospital transfers, were prevented from doing so for fear of viral transmission, even as home births increased due to the fear of hospitals as sites of contagion.

## Personal Protective Equipment (PPE) and Testing

Many of our articles indicate a profound lack of preparedness across hospitals and healthcare systems, epitomized by the initial lack—later remedied—of PPE. While hospitals first struggled and then found sufficient PPE for their providers, community-based providers and birthworkers struggled much longer to access PPE and testing (Gutschow and Davis-Floyd; Searcy and Castañeda). In many hospitals across the world, women in labor were forced to wear masks, despite the resultant hindrance of their ability to breathe heavily as needed. In contrast, many community-based midwives did not require laboring people to wear masks (Reyes; Gutschow and Davis-Floyd; DeYoung and Mangum; Benaglia and Canzini). Alonso et al. describe their search for the right kinds of masks and PPE that would afford them the fewest barriers to clients and the greatest ability to see clearly and work most effectively.

Several articles point to the exacerbated stressors that community-based midwives feel while attending to an increased load of clients in a context of asymptomatic community spread, without sufficient PPE and evidence about how to protect themselves or their clients (Crowther et al.; Alonso et al.; Gutschow and Davis-Floyd). In Puerto Rico, midwives face stereotypes that they are “‘dirty’, unsanitary, uneducated, and ill-equipped….that date back centuries…and are often associated with the race and ethnicity of the midwife,” while the lack of official status and government support leaves them vulnerable in times of medical crizes and shortages (Reyes). In contrast, New Zealand midwives have been inundated with requests for community-based care without proper governmental compensation, recognition, or reward for this increased client load (Crowther et al.).

## The Rise in Community Births

Several articles reported small but significant surges in community births—both at home and in free-standing birth centers—especially for women seeking to avoid the risks of hospital contagion and of separation from their support partners or newborns (Gildner and Thayer; Daviss et al.; Crowther et al.; Gutschow and Davis-Floyd). In an online survey of 980 women in the United States, 6% reported a new preference for community birth due to a desire for a more “natural” birth (Gildner and Thayer). *Motive matters*: some United States midwives reported that when pregnant women sought community birth simply out of fear of hospital contagion, and not out of an ideological commitment, those births might result in hospital transfers, leading some midwives to try to parse out individual motivations for seeking a community birth (Gutschow and Davis-Floyd). In the United States, some community midwives have been struggling to fully meet the increased demands for their services, for instance taking on as many as 8 births per month instead of the usual 4 (Gutschow and Davis-Floyd). While there were regions in the United States that reported dire shortages of community midwives to take on the increased demand, this was not the case in Canada and New Zealand. In those countries, government-certified midwives are fully integrated into the maternity care system and are trained in both home and hospital birth, making it far easier for them to adapt to shifts in site of birth (Crowther et al.; Daviss et al.).

Several of our articles show an increased divide between hospital-based and community maternity care during the pandemic, as the medical establishment fears and is threatened by the rise in community births and midwives’ power. In the United States, obstetricians denigrate home births with little evidence (Gutschow and Davis-Floyd; Daviss et al.), while in Puerto Rico, when demand for community births rose, obstetricians launched a ridiculously vicious campaign against the 24 community midwives on that island, who attend less than 1% of the more than 20,0000 annual births (Reyes).

## Turning to Traditional Midwives During COVID-19

Three articles that describe traditional midwives—in Kenya, Pakistan, and Guatemala—indicate a rise in community births as rural women fled hospital contagion to return to village midwives (Ali et al.; Ombere; Daviss et al.). Prior to the pandemic, traditional midwives attended 24% of all births in Pakistan, and 40% of all births in Kenya. In Totonicapán, Guatemala, before COVID-19, the *comadronas* were allowed to accompany their clients in the hospital and even to receive the baby, yet this beneficial practice was discontinued when the pandemic hit (Daviss et al.). The governments of Pakistan and Kenya have long attempted to restrict traditional midwives from attending births but rather to have them refer pregnant women to clinics or hospitals, yet these midwives continue to offer care to rural, underserved communities where there are few or no birth facilities. While the traditional midwives/*wakunga* of Kenya take COVID seriously (Ombere), some of the *Dāyūn* of Sindh Province, Pakistan consider COVID-19 to be a government plot to gain more foreign aid. The *Dāyūn* welcome the additional clients who seek their services due to fear of hospital contagion, while continuing to use their normal hygiene measures, such as washing their hands and keeping the birth space clean (Ali et al.).

Although the *comadronas* of Guatemala are well-trained by ICM standards, the traditional midwives of both Kenya and Pakistan admit that they need further training and resources from the government to reduce maternal mortalities and morbidities. Their low fees and reliance on traditional remedies like herbs, massages, and techniques for turning breech babies engender trust and support among the marginalized communities they serve. Yet instead of offering trainings to these traditional midwives, their governments push for 100% facility births while overlooking the deep gaps in access to or affordability of care, when families must pay on their own for essential medicines, supplies, and costs of transport (Ombere; Ali et al.; Gutschow et al., 2021).

We agree that traditional midwives should be better resourced and trained, more integrated into national or regional maternity care systems, and better integrated during referral or transfers of care to achieve a true continuity of care from home to hospital (Daviss and Davis-Floyd, 2021; Gutschow et al., 2021). Traditional midwives should be phased in, not out, in preparation for future pandemics or disasters in which their local, on-the-ground care will be needed, and to expand existing maternity care to underserved communities. Around 900,000 more skilled midwives are needed to reach full coverage of sexual, reproductive, maternal, newborn, and adolescent health (SRMNAH) needs (UNFPA, ICM, and WHO, 2021). At current rates of training and investment, it will take until 2030 to meet 80% of the SRMNAH needs, and the gap between the needs of low-income and high- or middle-income countries is expected to widen (UNFPA, ICM, and WHO, 2021).

## Fears and Stressors for Childbearers

Most of the article in this Special Issue report an amplification of fears and anxieties during the pandemic for childlbearers across widely disparate settings. The most common fears for childbearers include: fear of viral contagion, fear of being denied a labor support person, of having to choose between partner and doula, of being separated from their newborns, and of isolation during pregnancy, labor, and the postpartum period. These stressors have led to stalled labors, post-term births (Alonso et al.), miscarriages and stillbirths (Ozhiganova), and lower birthweight and preterm babies (DeYoung and Mangum). Although evidence is mounting, the rates of these complications and their full impacts on maternal and newborn health remain to be quantified. As facilities shifted their focus to tending to COVID-19 patients, childbearers were left struggling to access care, on top of the other traumas generated by the pandemic (DeYoung and Magnum; Rudrum; Gutschow and Davis-Floyd; Alonso et al.; Daviss et al.).

Many of our articles explore the lack of humanistic support for mothers, newborns, and families and the reversion to technocratic models of birth that ignore women’s rights and agency. DeYoung and Mangum theorize the rise of “disaster capitalism” to help explain the surge in infant formula offers shortly after birth, while other articles explore the ways in which COVID-19 has exacerbated underlying patterns of racism and hostility toward minoritized populations, including Indigenous, Black, and Brown communities (Oparah et al.; Rivera; Reyes; Daviss et al.; Crowther et al.). These articles confirm the value of community-based midwifery care in providing compassionate, respectful, and high-quality care within minoritized communities in times of crisis and normalcy.

## Racism and Inequities in Maternity Care

The articles in our collection illustrate the ways in which the pandemic has foregrounded inequalities, structural violence, and unequal risks of disease, death, and disability. As Reyes notes for Puerto Rico, the pandemic “is making more evident the extreme structural inequalities between the wealthy and the poor that already existed but are more visible now, and more severe...” Because many midwives, nurses, and doulas are women, their work and care are often devalued. As Crowther et al. explain:

Midwifery work is often not prioritized because relationships and care are not counted as measurable commodities and therefore get afforded less value… The risk of this focus is that it undervalues midwives’ significant emotional work of building and maintaining relationships. Yet it is established that relationships built and sustained over time enable intuitional ways of knowing that facilitate trust and safety…

The COVID-19 pandemic has had severe and disparate effects on birthworkers and women of color (Oparah et al.; Rivera; Reyes). Our articles confirm the value of community networks and knowledges that provide respectful and safe care for Black, Indigenous, and People of Color (BIPOC) in the face of ongoing racism and other social inequities. They note the importance of finding support for Black birthworkers and childbearers to mitigate and reduce the ongoing perpetration of varying forms of obstetric violence and neglect. Oparah et al. point out what other authors confirm: “For Black pregnant people, COVID-19 represents a crisis on top of a crisis: an already broken maternal health system attempting to deal with a life-threatening virus.”

In the United States, pregnancy-related mortality for Black women is three times that of non-Hispanic white women and quadruple that of Hispanic women; there is “ample evidence that these racial disparities in maternal outcomes are caused by the chronic stress of structural racism and providers’ implicit racial bias” (Gutschow and Davis-Floyd). Even in Canada, with its universal access to health care and widespread and well-integrated midwifery care, the pandemic exposed gaps in access that disproportionately affect First Nations and rural populations, who must often travel long distances to access life-saving maternity care (Rudrum; Daviss et al.).

## Provider Mistrust of Governments and Maternity Care Systems

Providers’ mistrust of both their governments and their maternity care systems is an emergent and salient theme. While Ozhiganova notes the high level of mistrust that Russian obstetricians have against their government, Leiva et al. describe the necessary resistance to the broad rules preventing partners in the labor room that the Chilean government promoted. In Mexico, government opposition to mask wearing early in the pandemic, even by the nation’s leading epidemiologist, was later overturned (Alonso et al.). While obstetricians in Russia resent the severity of top-down policies such as obfuscating and contradictory rules about hospital quarantine, severe infection control measures, and transporting mothers to different hospitals against their wills, they have been unable to openly resist for fear of punishment or backlash (Ozhiganova). While some Russian providers and hospitals adapted by accepting informal payments in order to unofficially admit labor support persons, this corruption does nothing to establish access to compassionate care for those who can’t afford such payments.

The deep mistrust in governments and maternity care systems that have imposed sharp restrictions on COVID+ laboring people has led childbearers in Russia, as in the United States, to disguise their COVID-19 symptoms (DeYoung and Magnum; Ozhiganova), while in Puerto Rico it was suspected that some maternal deaths were misrepresented as COVID-19 deaths (Reyes). Crowther et al. report governmental mistrust even among New Zealand midwives, who lack pay equity and whose needs are often ignored by their government, although 94% of New Zealand childbearers choose midwives as their primary caregivers. In the United States, Black birthworkers reported clients who, due to the pandemic, were not being seen in emergency rooms even when they were bleeding, or being sent home with no postpartum follow-up, despite having serious medical conditions (Oparah et al.).

## Telehealth and Virtual Ethnography

Our articles show that many obstetricians, midwives, and doulas resorted to telehealth during the pandemic for earlier prenatal visits. Obstetricians were particularly happy to use telehealth for prenatal visits, as they were “so timesaving” (Reyes; Gutschow and Davis-Floyd). For midwives, given that skilled physical touch is critical, for example, to determine fetal size and lie, most shifted to in-person visits later in the pregnancy. The use of telehealth for midwives and doulas can and did shift power differentials. Some midwives reported that childbearers were empowered by taking their own vitals and being responsible for their own health while learning from the midwives they met with virtually. Doulas became empowered to virtually access clients who might not be able to travel or afford doula care otherwise (Searcy and Castañeda; Rivera). Many doulas “were concerned about interjecting more technology into an already heavily technology-driven hospital” (Searcy and Castañeda). In the words of one South African doula, virtual doula care is “too much neocortex stimuli for the birthing person” (quoted in Searcy and Castañeda). Equity issues remain, as some clients who most need doula support may lack the necessary devices or the internet access required for virtual doula care. Furthermore, providers might shut down or disable their devices and thus sever the link between clients and doulas just when that link is most needed during labor or delivery (Searcy and Castañeda). In New Zealand, midwives seem to have worked out a judicious combination of telehealth and in-person appointments by generating “blended visits,” in which much of the appointment is conducted by telemedicine, leaving only those parts needed for a 15-min in-person consultation to limit possible viral exposure in indoor settings (Crowther et al.).

Two highly creative uses of telehealth were reported at La Florida Hospital in Santiago, Chile (Leiva et al.). Hospital staff created a text messaging system for department heads, on which they can quickly communicate with each other to make rapid adjustments to staffing schedules as they work to meet pandemic-created needs. Upon realizing the levels of stress pregnant clients were experiencing and their need for an open line of communication with hospital staff, staff members also created an Instagram account (Leiva et al.). This Instagram account, which is used to answer questions and offer virtual tours of the maternity ward, quickly gained a large following, as it allows pregnant and post-partum women to communicate 24/7 with a volunteer group of hospital midwives who can offer much needed solace in this time of confusion.

Because many countries responded to the COVID-19 pandemic with travel bans, ethnographers across the world were unable to access their field sites. Yet most of authors in this Special Issue found creative ways to engage in virtual fieldwork—conducting online research, virtual interviews, or digital surveys and questionnaires that produced excellent results. Oparah et al. demonstrate how such creative research can also advance the goals of participatory action research by insisting “on the interrelationships among theory, inquiry, reflection, and action, and re-imagining relationships between academic and community-based stakeholders in the research process.” They used “community-based sheltered-in-place research” by creating sharing circles in which Black birthworkers could share strategies for coping with the pandemic and structural racism, while finding community and creating safe spaces to speak and be heard. We urge our readers to examine the diverse and creative ethnographic research methodologies used by the authors who contributed to this Special Issue.

## Maternal and Newborn Rights During a Pandemic

Our articles cumulatively reveal the urgency of the need to protect women’s and newborn’s rights, which have been violated repeatedly in medical facilities, using the pandemic as an excuse. Such violations include enforced separation of mothers from newborns, mothers laboring alone without partners or other support persons, using “staff shortages” as excuses for neglecting mothers or newborns, lack of informed consent for rushed procedures, disrespectful care, and abusive care. Such violations have been harder to track and prevent due to the absence of labor support people, who would ordinarily have served as witnesses or deterrents against these types of abuse.

While La Florida Hospital in Santiago, Chile, which has been especially dedicated to humanistic care since its inception, has providers willing to push back against national policies that banned support people and were conceived without evidence (Leiva et al.), other hospitals that, pre-pandemic, had provided humanistic, woman-centered care, have found it extremely difficult to continue to do so. In the United States, bans on support people lasted for months in many hospitals (Gutschow and Davis-Floyd; Rivera), and there are still countries like Russia, Guatemala, and South Africa where women are routinely denied doulas, support people, and compassionate care, using COVID-19 as an excuse to again enact the principle of separation (Searcy and Castañeda; Daviss et al.; Ozhiganova).

In contrast, in settings that prioritize midwifery knowledges, relationships, and a respect for maternal agency, the outcomes are very different. In New Zealand, where nearly 15% of all births take place in a free-standing birth center or at home, the partnership of midwives with mothers and the importance of honoring Indigenous rights has led to more quickly identifying how restricting partners and families can lead to an abrogation of those rights (Crowther et al.). As in New Zealand, at the Luna Maya birth centers in Mexico and in community midwifery in the United States, including Puerto Rico, mothers’ rights to be with their newborns and their families or partners are preserved (Alonso et al.; Reyes; Gutschow and Davis-Floyd). At one hospital in Ottawa, Ontario, the Head of Pediatrics issued a letter clarifying the rights of mothers and newborns:

Parents have the legal and ethical right to make these decisions for their babies. At NO time do we have the right to remove a baby from its parents unless we have a legal order from the CAS (Children’s Aid Society), or if ethically the health professional is concerned for the baby’s well-being…in our recommendations to parents who are either suspects or COVID positive, it is very important to present the facts and known risks to their newborn baby so far. Since we have no evidence of harm to the baby if the mother wishes to skin-to-skin or breastfeed (with the precautions mentioned in the pandemic plan), *we cannot refuse this.* (Quoted in Daviss et al., emphasis theirs).

These examples indicate the possibility of protecting women’s and newborn’s rights and pursuing evidence-based protocols even in times of crisis, as well as the vulnerability of maternity care systems and the need for sustainable and resilient forms of maternity care (Gutschow et al., 2021). Our articles show that is possible to protect the rights of mother and newborn and to center clients wishes alongside provider’s recommendations even in times of disruption and crisis (Leiva et al.; Oparah et al.; Rivera; Alonso et al.; Gutschow and Davis-Floyd; Benaglia and Canzini).

Childbearers’ rights were at the forefront of birth activists’ activities in Italy, where activist Canzini wrote a letter to the Ministry of Health requesting the restoration of partners to the labor room. The letter explained why labor support was so critical for mothers, and helped to produce a policy shift in Bologna reversing the ban on labor support people (Benaglia and Canzini). Oparah et al. describe the virtual sharing circles that helped Black birthworkers find community and build on each other’s strategies in helping mothers self-advocate, including for the right to doula care and to room in with their newborns. One United States midwife reported that her most significant lesson learned from COVID was that: “groups of people and organizations CAN work together quickly and effectively in the interest of public health” (quoted in Gutschow and Davis-Floyd).

## The Need for Full Integration of Community Birth Providers

One of the primary needs expressed in all of the articles that cover community birth providers is the need for their full integration into their country’s health care systems. With the exceptions of the Netherlands, New Zealand, and Canada, the majority of the world’s community-based midwives, including traditional midwives, are left with little or no governmental support in ways that are detrimental to their practices, their training, their morale, and their clients.

There are many problems with lack of integration for community-based midwives. From the provider’s side these can include: lack of insurance, lack of physician backup and of smoothly functioning referral systems in case of complications, and the lack of respect for midwives and their clients shown during transfers of care. For childbearers, the issues include lack of access to care in rural or underserved communities and rude and disrespectful treatment in facilities. Most of these barriers can be overcome if midwives and their clients are treated well upon arrival, and the community midwives are allowed to stay in the hospital to provide labor support and continuity of care to their clients.

In the United States, the issue of transport was addressed in a national summit, during which Best Practice Guidelines were developed (Gutschow and Davis-Floyd). While these Guidelines have been widely disseminated and many community midwives try to follow them, many hospitals still ignore them. Where such guidelines are lacking and where home births are more marginalized or even illegal, community midwives and homebirth obstetricians are often persecuted until they are pushed out of practice.

Because some women around the world will seek midwife-attended births at home or in birth centers regardless of legality, we call for the legalization and integration of community midwives—including traditional midwives—into maternity care systems across the globe. This would require legalizing and licensing them in all countries, offering them insurance coverage where needed, respecting their services during referral and transport to hospitals, and adequately compensating them for their services. *All of our articles dealing with community birth echo these points*. To keep community midwives on the fringes of the healthcare system reflects outdated ill-will from hospital-based providers who may not welcome the competition—but causes great harm.

A recent report on midwifery across the globe notes the urgent global need for professional midwives in order to achieve 100% coverage of sexual, reproductive, maternal, newborn, and adolescent health (SRMNAH) care (UNFPA, ICM, and WHO, 2021). While the total healthcare shortage for the SRMNAH workforce is 1.1 million, roughly 90% of the shortage comprises midwives, who provide services that are more efficient, cost-effective, and accessible than physicians in many rural, low-income settings. The costs savings that would result from midwifery care outside hospitals are enormous. The United States alone would save roughly *$11 billion* if only 10% more births took place in homes and freestanding birth centers (Daviss et al.). While the safety of planned, midwife-attended community births for low risk mothers is well-proven, some providers continue to insist, without evidence, that hospital birth are always safest. Yet they are not always safest for low risk births (ibid.) And facility-based births without skilled providers or essential medicines and technologies can entail maternal and newborn morbidities and mortalities, even for low-risk mothers (Miller et al., 2016; Gutschow et al., 2021).

The rise in community births resulting from fears during COVID-19 has highlighted the fact that *healthcare systems can no longer ignore the desire for community birth with midwives.* In times of crises, *decentralized* care can be more efficient and accessible for patients and providers, who are working to decrease inequities and structural violence in their healthcare systems (Renfrew et al., 2021). In Puerto Rico, which was hit by two hurricanes in recent years, a midwifery disaster response would include:

planning for emergency care by mapping the location of midwives, supplying them with basic equipment and medications, and legitimizing their profession with an appropriate scope of practice, licensing, back-up, and incentives (Reyes).

To preserve and continually enhance their midwifery skills, community midwives need government support, ongoing training, certification, and again, full integration into their respective healthcare systems. These would begin to remove the many barriers to their practices and aid their expansion into settings where they are most needed. In hospitals, there is a huge need for midwives who practice the midwifery model of care and who are recognized as *colleagues/*equal partners by obstetricians, rather than as subordinates, as is often the case (Davis-Floyd, 2018b). We hope that providers, maternity care systems, and activists alike will seize COVID-19 as a moment to move toward positive change, rather than continuing to entrench harmful and non-evidence-based care (Gutschow et al., 2021).

## Conclusion: The Pandemic as a “Touchstone” and a “Pivotal” or “Transformational” Moment

Some of the articles in this Special Issue term the pandemic a “pivotal,” “transformational,” “touchstone,” or “watershed” moment that has revealed and highlighted syndemic deficiencies and disparities in multiple national maternity care systems. Many of our articles show the fragility of efforts to honor reproductive rights, the invisibility of midwifery care that is often undercompensated, and the resistance to humanistic changes that characterizes many industrialized or technocratic maternity care systems (Davis-Floyd, 2003; Gutschow et al., 2021).

With the arrival of vaccines in many countries, the global coronavirus pandemic will change shape in ways that cannot be fully predicted. In hindsight, we wish to stress the ways in which COVID-19 has helped to identify and make visible the multiple disparities that produce suffering and harm, especially for BIPOC, rural, and other marginalized communities. We emphasize the need for positive change and the disruption of dysfunctional habits during an already disruptive pandemic. We hope that birthworkers, researchers, and policy makers will recognize this pivotal moment as an opportunity for humanistic change. If we are to succeed, we must continue to call out structural disparities and dysfunctions, while maternity care providers and policy-makers will need to respond.

Let us hope that the COVID-19 pandemic will facilitate critical changes in systems and practices. We hope that key lessons from the pandemic—limiting unnecessary interventions, providing continuous labor support, immediate skin-to-skin contact, and breastfeeding—can be preserved so as to improve outcomes for mothers and newborns. We trust that providers, too, will benefit from these humanistic changes and improved working conditions. We would be heartened to see the principles of separation and prohibition fade away and be replaced by the fundamental principles of connection, agency, and human rights in childbirth for all.
